# Evidence-Based Treatment Algorithm for Freiberg Disease

**DOI:** 10.1177/19476035231205676

**Published:** 2023-10-10

**Authors:** Ichiro Yoshimura, Masato Takao, Emilio Wagner, Sjoerd Stufkens, Jari Dahmen, Gino M.M.J. Kerkhoffs, Mark Glazebrook

**Affiliations:** 1 Faculty of Sports and Health Science, Fukuoka University, Fukuoka, Japan; 2 Clinical and Research Institute for Foot and Ankle Surgery, Chiba, Japan; 3 Departamento de Traumatologia, Clinica Alemana-Universidad del Desarrollo, Santiago, Chile; 4 Department of Orthopedic Surgery, Amsterdam UMC, University of Amsterdam, Amsterdam, The Netherlands; 5 Reconstructive Foot & Ankle Surgery and Orthopedic Sports Medicine, Queen Elizabeth II Health Sciences Center, Halifax, NS, Canada

**Keywords:** Freiberg disease, surgery, review

## Abstract

Freiberg disease is a type of osteonecrosis of the metatarsal head that predominantly occurs in young females and adolescents, although it may occur at any age. The pathophysiology is multifactorial and may involve trauma, altered foot biomechanics, systemic disorders, and arterial insufficiency. The most typical location is the second metatarsal head, but Freiberg disease may also occur in other lesser toes. Nonoperative treatment is best applied in the early stage of the disease; if this is ineffective, surgical treatment is recommended. Currently available surgical procedures include debridement, osteotomy, osteochondral grafting, microfracture, interposition arthroplasty, implant arthroplasty, and metatarsal shortening arthroplasty. In this article, we propose a treatment algorithm for Freiberg disease based on the current literature and expert opinion.

## Introduction

Freiberg disease is a type of osteonecrosis of the metatarsal head and may occur in people of all ages; however, it typically occurs from adolescence through the second decade of life.^1-3^ Freiberg disease is more common in females, especially those in the athletic population.^4^ This condition was first reported in 1914, when Freiberg^1^ described 6 cases of infraction of the second metatarsal head.^5,6^ The author suggested that minor trauma to the foot was associated with onset of the disease. This condition results in sclerosis, flattening, and collapse of the metatarsal head with subsequent osteoarthritis of the metatarsophalangeal (MTP) joint, and it is currently known as Freiberg disease. It is generally accepted that the cause of Freiberg disease is multifactorial and may involve minor/major trauma, abnormal foot biomechanics, and hypovascularity.^3^ In general, nonoperative management is the first-line treatment for symptomatic Freiberg disease. When nonoperative treatment is ineffective, surgical treatment is indicated. However, various surgical procedures have been described in small case series.^7-9^ Thus, no clear algorithm for surgical treatment decisions has been established. The present review was performed to provide up-to-date information on the treatment of Freiberg disease based on current articles and specialists’ consensus to assist in making treatment decisions.

## Classification and Staging

**Figure 1.**
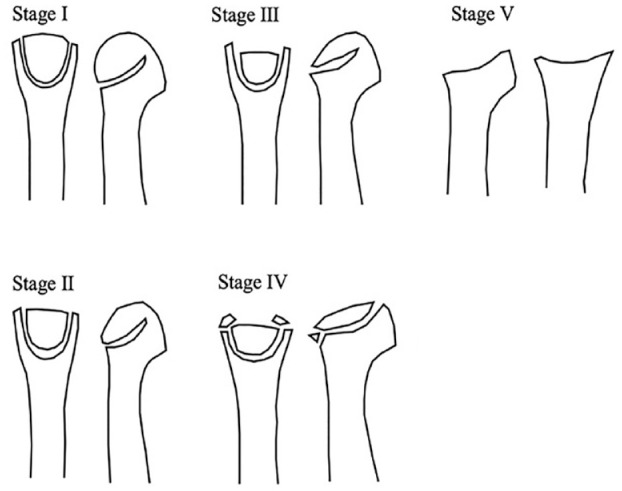
Smillie classification. Stage I: A narrow fissure fracture in an ischemic epiphysis. Stage II: Absorption of cancellous bone in the metatarsal head with sinking of the articular surface dorsally. Stage III: Further absorption and sinking of the articular surface with larger projections on either side. Stage IV: Deeper sinking of the articular surface with peripheral projections/fractures. Stage V: Degenerative arthrosis with flattening and deformity of the metatarsal head.

Several articles have suggested staging schemes for Freiberg disease. The most widely adopted classification is Smillie’s classification system, which is based on surgical findings.^10^ This staging system is useful as a classification tool to determine the current disease progression. For more accurate staging, it is suggested using it on computed tomography (CT) images (**Fig. 1**):

- Stage I: A narrow fissure fracture in an ischemic epiphysis with sclerosis between cancellous surfaces.
- Stage II: Absorption of cancellous bone in the metatarsal head with sinking of the articular surface dorsally and no involvement of the plantar aspect.
- Stage III: Further absorption and sinking of the articular surface with larger projections on either side.
- Stage IV: Deeper sinking of the articular surface with peripheral projections/fractures. Restoration of the normal articular surface is impossible.
- Stage V: Degenerative arthrosis with flattening and deformity of the metatarsal head. The plantar aspect of the metatarsal head is involved. The shaft of the metatarsal bone is thickened and dense.

## Nonoperative Management

Nonoperative treatment is the first-line treatment for Freiberg disease.^7^ The aim of nonoperative treatment is to relieve pain and minimize metatarsal head deformation.^8^ Primary management of Freiberg disease involves nonoperative treatments such as pain medications, activity modification, immobilization, shoe wear modifications, orthotics, and other modalities.^11^ Nonoperative treatment is reportedly most successful in the early stages, with 60% efficacy.^12^ Helal and Gibb^13^ reported that most patients with stage I and II Freiberg disease responded to nonoperative treatment and could be expected to have long-term success. Thus, patients with minimal metatarsal deformity (stages I and II) are considered good candidates for nonoperative treatment. If the patient’s condition does not respond to nonoperative treatments, then surgical treatment is considered.

## Surgical Management

**Figure 2.**
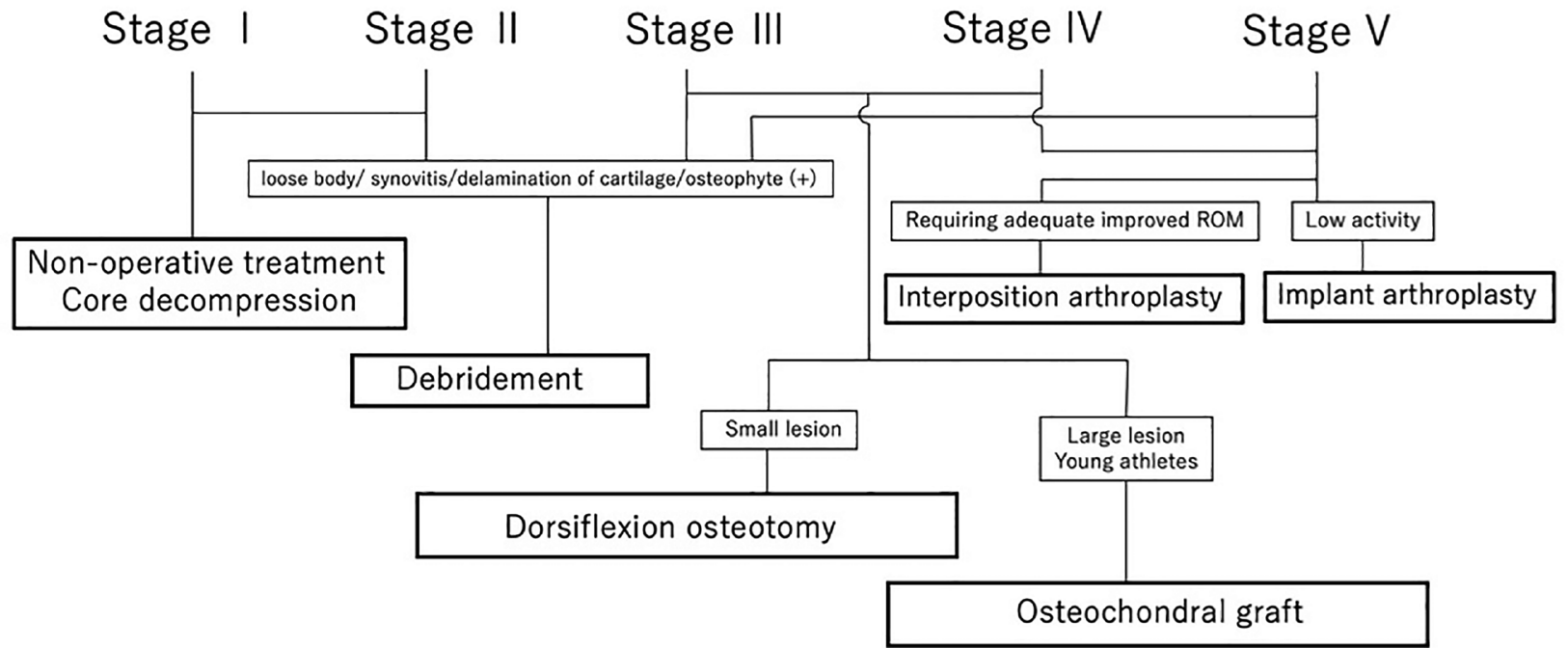
Treatment algorithm of Freiberg’s disease.

Patients with Smillie’s stage I and II Freiberg disease usually undergo nonoperative management for at least 6 months. Surgical management should be reserved for patients who do not respond to nonoperative management. Various surgical procedures exist and can be divided into joint-preserving procedures and joint-sacrificing procedures.^13^ Joint-preserving and joint-sacrificing procedures are performed in the early and late stage, respectively, based on the above-described classification system **(Fig. 2)**.

## Joint-Preserving Procedures

### Core Decompression

Surgical core decompression has been successfully utilized for avascular necrosis of the femoral head/talus.^14,15^ This procedure is intended to reduce the elevated interosseous pressure and allow for revascularization of the necrotic region. Two case reports described the core decompression procedure for early-stage Freiberg disease.^16,17^ A Kirschner wire was used to create multiple holes from the articular surface to the metatarsal head through the dorsal approach. Both case reports described resolution of symptoms with no internal structural changes of the metatarsal head. The core decompression procedure usually causes no structural changes to the metatarsal head in early-stage disease.

### Open Joint Debridement

**Figure 3.**
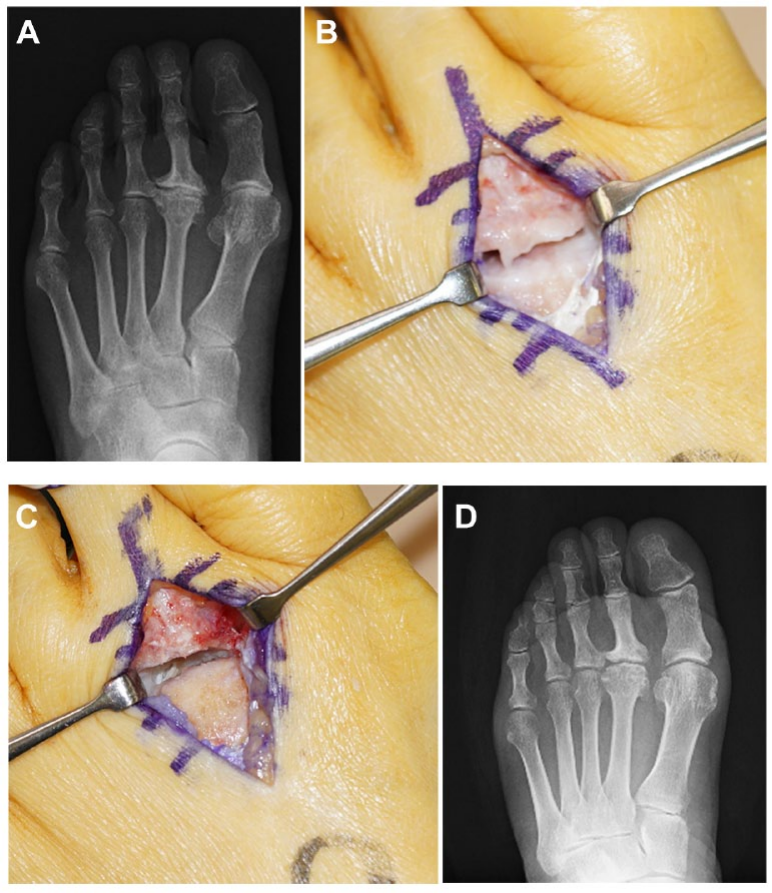
Open debridement. (**A**) Osteoarthritis in second metatarsophalangeal (MTP) joint by Freiberg disease. (**B**) Preoperative radiograph. Joint space narrowing and osteophyte formation were observed in the second MTP joint. (**C**) After removal of the osteophyte and synovium. (**D**) Postoperative radiograph. Widening of the MTP joint were observed after debridement.

Joint debridement is a simple procedure that can be adopted at any stage of Freiberg disease. This procedure includes removal of synovium, loose bodies, osteophytes, and delaminated articular cartilage **(Fig. 3)**.^17-21^ The open debridement procedure was originally proposed by Freiberg^12^; later, Sproul *et al*.^20^ reported the clinical outcomes of open debridement in 11 athletes. All patients showed improvement of their symptoms with restoration of 80% of their normal range of motion. Erdil *et al*.^21^ showed that debridement for Freiberg disease resulted in significant improvement of clinical outcomes and a better self-reported health status.

### Arthroscopic Joint Debridement

Three case reports have described arthroscopic debridement of Freiberg disease.^22-24^ Maresca *et al*.^22^ performed arthroscopic debridement and drilling of the metatarsal head for a 28-year-old patient with bilateral Freiberg disease (Smillie stage II). At 2 years after surgery, the patient was free of symptoms and magnetic resonance imaging showed no destruction of the metatarsal head. Carro *et al*.^23^ reported arthroscopic debridement and resection of the base of the proximal phalanx in a 60-year-old man with Smillie stage IV Freiberg disease. At 2 years postoperatively, the patient was symptom-free. Lee *et al*.^24^ performed arthroscopic procedures (removal of loose bodies, synovectomy, and drilling of the metatarsal head) for a 36-year-old-woman with a Smillie stage IV lesion. At 2 years postoperatively, the patient was symptom-free and radiographic examination showed satisfactory improvement. Currently, it is appropriate to perform arthroscopy as an adjuvant treatment following other established procedures.

### Metatarsal Osteotomy

**Figure 4.**
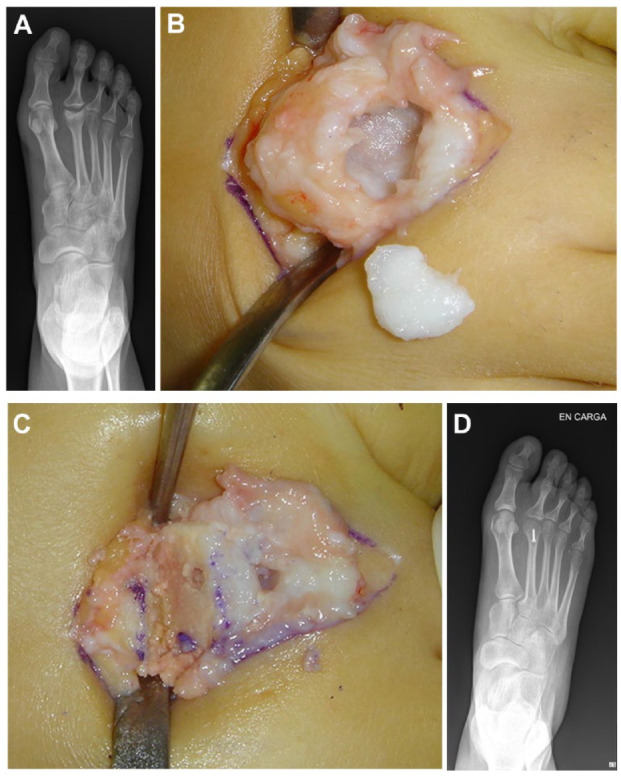
Dorsiflexion osteotomy. (**A**) Collapse of the articular surface in the second metatarsal head on a radiograph. (**B**) Intraoperative condition of the second metatarsal head: osteochondral fragment and metatarsal head. (**C**) After excision of the damaged area in the metatarsal head. (**D**) Closing wedge to bring up and fix using metal screw.

Two different types of osteotomies have been reported for Freiberg disease: shortening metatarsal osteotomy and dorsiflexion osteotomy (DFO). Shortening osteotomy achieves offloading of the metatarsal head. The purpose of DFO is to replace the joint surface and decompress the metatarsal head **(Fig. 4)**.

Smith *et al*.^25^ reported the clinical outcomes of extra-articular metatarsal shortening osteotomy. Fourteen of 15 feet achieved pain relief within 12 months after the operation. The metatarsal bone was shortened by approximately 4 mm, and stabilization was performed with a dorsal T-plate. However, 4 feet showed decreased flexion, and the toes did not contact the ground.

In 1979, Gauthier and Elbaz^26^ described DFO in 53 cases. The DFO included osteotomy at the metatarsal head, excision of the damaged area, and a closing wedge to bring up the healthy joint cartilage. Many articles have described the operative techniques and reported good clinical outcomes.^25-31^ DFO is one of the most well-known joint-preserving procedures. Chao *et al*.^27^ performed DFO for 13 patients and achieved excellent results in 4 and good results in 7 without postoperative complications. Lee *et al*.^28^ performed DFO using an absorbable pin with an average follow-up of 45 months (range, 22-84) months. The range of motion of the MTP joint was improved by 26 degrees after osteotomy. The mean metatarsal length was shortened by 1.7 mm (range, 1-2 mm). The visual analog scale (VAS) score decreased from an average of 8.0 to 2.3 (*P* < 0.05). All patients were satisfied with the surgical outcome.

Helix-Giordanino *et al*.^30^ reported a case series of 30 patients with an average follow-up of 6.5 years. The mean American Orthopedic Foot & Ankle Society (AOFAS) score was 83.8 ± 11.8 points (17 patients, very satisfied; 11 patients, satisfied; 2 patients, moderately satisfied), and the average shortening was 2.0 ± 1.4 mm.

Pereira *et al*.^31^ reported the long-term results of DFO. The mean follow-up period was 23.4 (range, 15-32) years. The clinical outcomes were classified as excellent in 80% of cases and good in 20%. The mean AOFAS score was 96.8 (range, 91-100). There was a strong negative correlation between the Smillie’s classification and the AOFAS score (r = −0.88, *P* < 0.001). Many articles have described favorable clinical outcomes after DFO. However, Chao *et al*.^27^ reported an average 15° decrease in range of motion in the MTP joint after DFO. This occurred because the procedure included bringing up the normal plantar articular surface dorsally.

Dorsiflexion osteotomy is indicated for Smillie stage III and IV Freiberg disease, and a small lesion is preferred to avoid a decrease in range of motion after DFO.

### Osteochondral Autologous Transplantation

**Figure 5.**
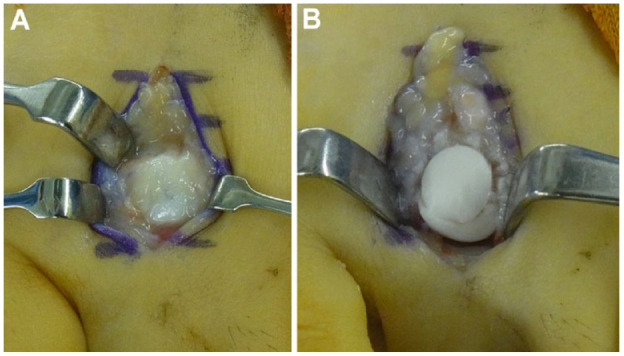
Osteochondral autologous transplantation. (**A**) Collapse of articular surface of second metatarsal head. (**B**) The cartilage surface was reconstructed with the osteochondral plug.

The first report of osteochondral autologous transplantation (OAT) for Freiberg disease was described by Hayashi *et al*.^32^ in 2002. The advantages of this procedure include restoration of the joint surface with hyaline cartilage, achievement of a normal bone complex close to the normal anatomical configuration, and early bone-to-bone healing. This procedure consists of 3 steps: creation of a hole at the metatarsal head, including the degenerative area; harvesting of the osteochondral autograft plug from the ipsilateral knee; and press-fit transplantation of the plug without fixation into the prepared hole of the metatarsal head **(Fig. 5)**. Tsuda *et al*.^33^ performed OAT for 3 adolescent athletes with Smillie’s stage III/IV Freiberg disease. In all 3 cases, a return to the original sports activities was achieved within 3 months. No patients reported donor-site pain or discomfort. OAT has provided great advantages for student athletes who can only play for a limited period of time in junior high school and high school.

Miyamoto *et al*.^34^ reported the midterm results of OAT for advanced-stage Freiberg disease (stage III/IV), showing improvements in the mean AOFAS and VAS scores as well as improvements in dorsiflexion and plantar flexion range of motion. Ishimatsu *et al*.^35^ investigated the outcomes of OAT in 12 young athletes. They reported significant improvements in the Halasi score and Japanese Society for Surgery of the Foot score. All patients returned to sport activities at a mean of 3.5 months after surgery, and the range of motion of the MTP joint significantly improved.

Articles with detailed comparisons of OAT and DFO have been published. Georgiannos *et al*.^36^ performed a randomized controlled trial in which the mean AOFAS score improved significantly in the DFO and OAT cohorts. However, the patients in the OAT group returned to sports significantly earlier than those in the DFO group. Osteochondral autologous transplantation accelerates return to the original sports level for 2 reasons: (1) it improves the range of motion and conformity of the joint and (2) it provides a large contact area of the cylindrical surface and early bone-to-bone healing.

Kim *et al*.^37^ compared OAT and DFO among adult patients with late-stage Freiberg disease. The mean AOFAS score was significantly better in the OAT group, and the plantar flexion angle was significantly lower the DFO group.

The OAT procedure appears to be equal to or better than DFO in terms of the clinical outcome, range of motion of the MTP joint, complications, and return to sports; however, the occurrence of knee donor-site morbidity after OAT remains a concern. Shimozono *et al*.^38^ recently performed a meta-analysis of knee-to-talus donor-site morbidity after OAT. The estimated proportion of donor-site morbidity ranged from 6.7% to 10.8%, and the authors concluded that the estimates of knee donor-site morbidity may be lower than previously considered.

Osteochondral autologous transplantation is indicated for patients with large stage III and IV lesions who are expected to have a quick return to sports activities. Young athletes are particularly good candidates for OAT.

## Joint-Sacrificing Procedures

### Interposition Arthroplasties

It is difficult to restore the metatarsal head and improve symptoms in patients with later stages (Smillie’s stage IV/V) of Freiberg disease. Resection arthroplasty provides good clinical outcomes for patients with inflammatory arthropathy. Over time, however, patients may develop joint space narrowing and residual pain and deformity of the lesser toes. Soft tissue interposition arthroplasty has been recommended for late-stage Freiberg disease.^3^ Several articles have reported good clinical outcomes of interposition arthroplasty using soft tissue such as the extensor digitorum longus, extensor digitorum brevis, palmaris longus, periosteum with a fat pedicle graft, and allograft of the semitendinosus tendon.^39-44^

Çevik *et al*.^44^ reported the mid- to long-term results of interposition arthroplasty with the extensor digitorum brevis tendon. After a mean follow-up 133.8 (range, 60-198) months, the AOFAS scores improved as did the range of motion in dorsiflexion and plantar flexion. No patients showed joint space narrowing on radiographs.

Abdul *et al*.^42^ reported the functional outcomes of local pedicle graft interposition arthroplasty. Twenty-three patients underwent interposition arthroplasty using a periosteum and fat pedicle graft from the metatarsal shaft. The mean follow-up was 3.5 years. A total of 35% of patients returned to normal footwear, 43% of patients returned to fashion footwear, and 22% of patients returned to sports activities. The AOFAS and VAS scores also improved. Stautberg *et al*.^43^ reported the clinical and radiographic outcomes of interposition arthroplasty with improvements in the VAS score. The Foot and Ankle Ability Measure sports subscale score also significantly increased, and 90% of patients reported their function as normal or nearly normal after surgery. Radiographically, the ratio of the affected metatarsal length to the adjacent metatarsal length was maintained after surgery.

Interposition arthroplasty provides good outcomes for patients with late-stage (stage IV/V) Freiberg disease.

### Implant Arthroplasty

In the end stage of MTP joint osteoarthritis, some patients undergo replacement arthroplasty with an interposition spacer/separate articulating implant. Cracchiolo *et al*.^45^ performed silicone implant arthroplasty for degenerative MTP joint disorders, including 6 cases of end-stage Freiberg disease. Sixty-three percent of feet had good results at the 3-year follow-up. Four of the 6 feet had good results and 2 feet had reservations. The reasons for the reservations were transfer metatarsalgia in 1 foot and stress fracture in the other. In addition, the silicone implant developed complications including breakage and silicone synovitis. Townshend and Greiss^46^ performed total ceramic arthroplasty for 9 patients with destructive disorders of the lesser MTP joints, including Freiberg disease. The mean follow-up was 23 (range, 6-46) months, and the mean postoperative AOFAS score was 75 (range, 42-92) points. Prosthesis failure occurred in 1 patient. Glazebrook *et al*.^47^ presented their preliminary experience with Cartiva (Carticept Medical, Alpharetta, GA, USA), a polyvinyl alcohol hydrogel implant for pathology of the second metatarsal head. The results demonstrated good outcomes and no complications in 5 patients (mean age, 57 years) at a mean follow-up of 25 months.

Implant arthroplasty is indicated in limited cases with low activity of late-stage Freiberg disease and is not recommended as routine treatment.

## Summary

Freiberg disease is a rare disorder that presents with a variety of symptoms in patients of a wide age range but especially in young people. Nonoperative therapy is generally most suitable for early-stage disease; however, if nonoperative therapy is ineffective for more than 6 months, surgery can be considered appropriate. There is no clear algorithm of surgical treatment because of the limited number of previous articles. We propose a treatment algorithm for Freiberg disease based on the current literature and expert opinion. The choice of surgical procedure should be based on a shared decision making model, encompassing both patient and lesion characteristics. This article provides a best evidence available, clinical decision-making tool to assist in adopting a specific treatment option for each stage of Freiberg disease.
